# Ophthalmological emergencies and the SARS-CoV-2 outbreak

**DOI:** 10.1371/journal.pone.0239796

**Published:** 2020-10-01

**Authors:** Chiara Posarelli, Maria Novella Maglionico, Giuseppe Covello, Pasquale Loiudice, Alessandro Cipriano, Massimo Santini, Michele Figus

**Affiliations:** 1 Ophthalmology, Department of Surgical, Medical, Molecular Pathology and of Critical Care Medicine, University of Pisa, Pisa, Italy; 2 Emergency Department, Azienda Ospedaliero-Universitaria Pisana (AOUP), Pisa, Italy; 3 Emergency Medicine and Emergency Room Unit, AOUP, Pisa, Italy; National Institute for Infectious Diseases Lazzaro Spallanzani-IRCCS, ITALY

## Abstract

Since the end of 2019, an outbreak of the severe acute respiratory syndrome coronavirus 2 (SARS-CoV-2), originating in the Chinese city of Wuhan has spread rapidly worldwide causing thousands of deaths. Coronavirus disease (COVID-19) is supported by SARS-CoV-2 and represents the causative agent of a potentially fatal disease that is of great global public health concern. Italy has been the first European country recording an elevated number of infected forcing the Italian Government to call for total lockdown. The lockdown had the aim to limit the spread of infection through social distancing. The purpose of this study is to analyze how the pandemic has affected the patient’s accesses to the Ophthalmological Emergency Department of a tertiary referral center in central-northern Italy, during the lockdown period. The charts of all patients that came to the Emergency Department during the lockdown period (March 10 –May 4, 2020) have been retrospectively collected and compared with those in the same period of 2019 and the period from 15 January– 9 March 2020. A significant reduction of visits during the lockdown has been observed, compared with those of pre-lockdown period (reduction of 65.4%) and with those of the same period of 2019 (reduction of 74.3%). Particularly, during the lockdown, minor and not urgency visits decreased whereas the undeferrable urgency ones increased. These pieces of evidence could be explained by the fear of patients to be infected; but also revealed patients misuse of emergency services.

## Introduction

Dr. Li Wenliang, a 34-years-old Ophthalmologist, was the first to presage the international recognition of the current pandemic crisis over three weeks [1]. On the 7^th^ of February 2020, he died in Wuhan Central Hospital (China) due to the respiratory complications related to a novel Coronavirus (nCOV) [1]. Since the end of 2019, an outbreak of the SARS-CoV-2, originating in the Chinese city of Wuhan has spread rapidly worldwide causing thousands of deaths.

The virus has been identified as the causative pathogen of a disease called COVID-19 which can cause mild sickness or be asymptomatic for most patients but which can also be responsible for severe respiratory failure and death [2].

COVID-19 has been declared officially pandemic on the 11^th^ of March 2020 with over 5 million people affected [3].

In February 2020, the coronavirus epidemic reached Italy with the first cases registered in the city of Codogno, Lombardia. The Italian Government aiming to limit the infections and the spread of the virus ordered a national lock-down. People were forced to stay at home except for work, urgent matters or health reasons. All the commercial and productive activities, excluding those who provided essential services, were closed [4].

The Italian outbreak brought all clinical activities to a long period of standstill. In our hospital all the appointments and elective surgeries were cancelled and, in order to respond to the needs of many infected patients, most departments were converted to COVID-19 Emergency Units.

The only guaranteed services were the emergencies. We have tried to provide care at home through telemedicine but this kind of service is suitable for chronic patients or for viewing and commenting on instrumental exams, but not for patients who are acutely ill [5, 6].

As already observed in the early 2000s during the SARS epidemic, the fear of contagion may affect the demand for hospital services and several editorials published in recent months have already reported a dramatic change in the healthcare access [7–9].

COVID-19 probably has instilled the fear of face-to-face medical care and also the ophthalmological consultations decreased [8, 10, 11] leading physicians to ask: “Where did all the ophthalmological emergencies go?”.

In this study, we aimed to assess how the pandemic and the people’s fear of being infected has modified the accesses to the Ophthalmological Emergency Department of Azienda Ospedaliero-Universitaria Pisana (Pisa, Italy) during the lockdown.

## Materials and methods

In this retrospective, single-center, observational study, we analyzed the records of all subjects referring to the Ophthalmological Emergency Department of Azienda Ospedaliero-Universitaria Pisana, during the period from March 10, 2020, that is the day in which the Italian minister decreed a national lockdown for most activities except essential ones [4], to May 04, 2020, the beginning of phase 2 with the gradual reopening of activities [12]. We compared the data of the lockdown period with those of the same period (10 March—04 May) of 2019 and with those of the 55 days of the pre-lockdown period (15 January– 9 March) of 2020. The total number of visits, patient’s demographics, date of admission, diagnosis, emergency code, type of discharge were retrieved. We further collected the number and the reason for ophthalmological consultations requested by the emergence department or other hospital wards. Diagnoses were categorized using codes from the International Classification of Diseases, Ninth Revision, Clinical Modification (ICD-9- CM).

We divided the conditions diagnosed in 5 categories (emergency, undeferrable urgency, deferrable urgency, minor urgency and not urgency). These categories were assigned at discharge by the ophthalmologist of the emergency department.

We further subdivided diagnosis into 10 subclasses: orbit and annexes, anterior segment, visual disturbance, lens, vitreous, retina and choroid, neuro-ophthalmology, trauma, inflammation, other.

This study was approved by the Ethical Committee Area Vasta Nord-Ovest (CEAVNO) in Pisa (KE-17826/117/2020). The study design was conducted according to the principles of the Declaration of Helsinki (1975) and its revised version of 2013. Data were anonymized for collection and analysis, avoiding a privacy data breach.

Statistical analysis was performed using SPSS software version 25 for Windows (IBM Corporation, Armonk, NY, USA). Descriptive statistic was used to summarize mean values and standard deviations of all numerical data. Independent sample t-test and χ2 test were used to compare continuous and categorical variables, respectively. P values < 0.05 were considered significant.

## Results

The total number of visits during the lockdown period (10 March– 04 MAY 2020) was 246 and was markedly reduced if compared with the pre-lockdown period (711 cases) and with the same period of 2019 (959 cases). We observed a reduction of 65.4% if compared with the pre-lockdown period and a reduction of 74.3% if compared with 10 March– 04 May 2019 period.

The male / female proportion of lockdown period (147 males and 99 females) did not significantly change if compared with the same period of 2019 (526 males and 433 females, *P* = 0.172) and with the pre-lockdown period (383 males and 328 females, P = 0.303).

**Fig 1:**
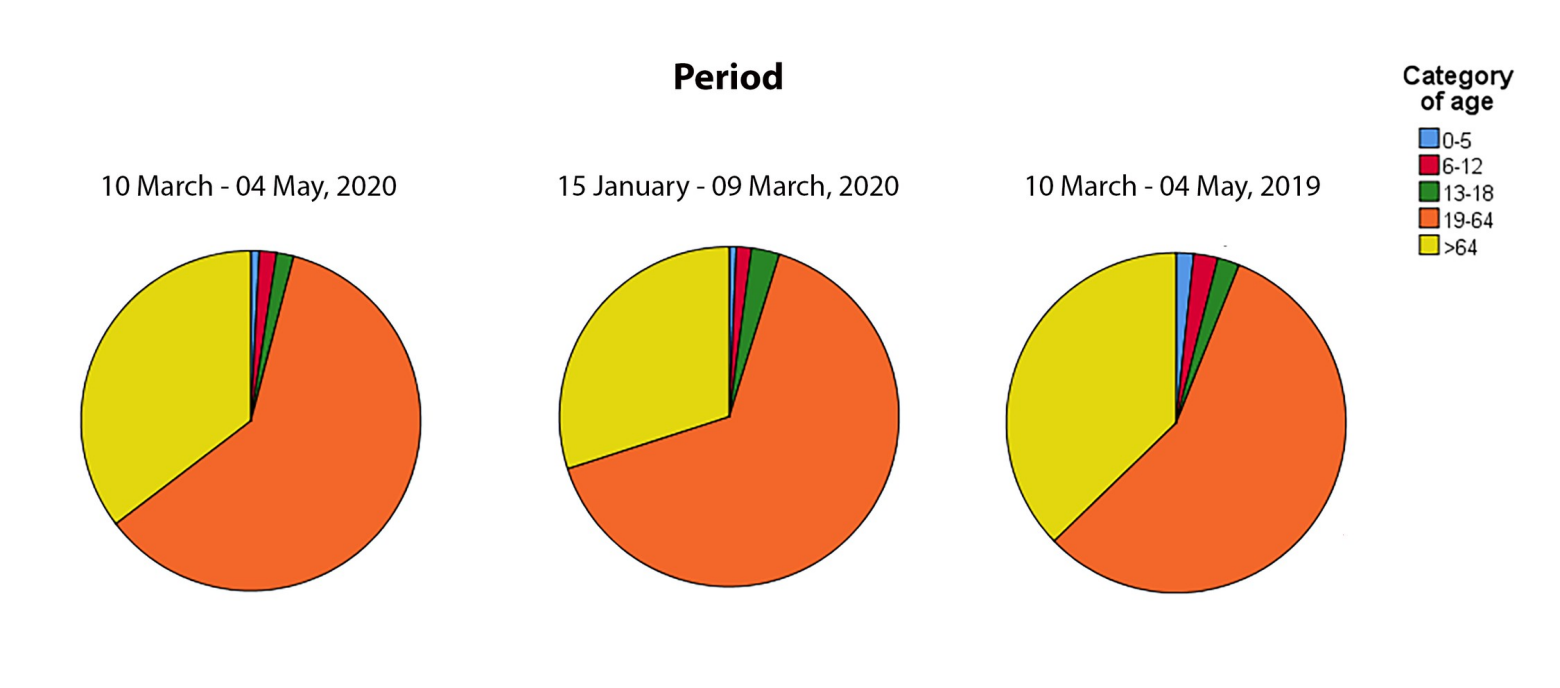
Number of visits categorized by age. Pie charts display the number of visits performed at the Ophthalmological Emergency Department during the 2020 lockdown period (from March 10 to May 10), compared with the same period of 2019 and with the pre-lockdown of 2020 (from January 15 to March 09).

The mean age of patients during the lockdown period was 55.5 ± 18.3 years and did not significantly change in the pre-lockdown period of 2020 (53.1 ± 19.4 years, *P =* 0.083) and with the 10 March– 04 May period of 2019 (53.7 ± 21.1 years, *P =* 0.226). Table 1 and Fig 1 display the number of visits categorized by age.

**Table 1:** Number of visits categorized by age.

Age category	10 MAR– 04 MAY 2020	15 JAN– 09 MAR 2020		10 MAR– 04 MAY 2019	
	Frequency (%)	Frequency (%)	*P value*^a^	Frequency (%)	*P value*^b^
0–5	2 (.8)	5 (.7)	.862	16 (1.7)	.329
6–12	4 (1.6)	10 (1.4)	.807	22 (2.3)	.528
13–18	4 (1.6)	19 (2.7)	.366	20 (2.1)	.651
19–64	149 (60.6)	464 (65.3)	.533	544 (56.7)	.574
>64	87 (35.4)	213 (30.0)	.259	357 (37.2)	.712
Total	246	711		959	

Number of visits categorized by age performed at the Ophthalmological Emergency Department during the 2020 lockdown period (from March 10 to May 10), compared with the same period of 2019 and with the pre-lockdown of 2020 (from January 15 to March 09). The number of visits is presented in absolute and percentage values (between round brackets).

*P value*^a^ = lockdown period (10 MAR– 04 MAY 2020) compared with pre-lockdown period (15 JAN– 09 MAR 2020), χ^2^ test

*P value*^b^ = lockdown period (10 MAR– 04 MAY 2020) compared with the same period of 2019, χ^2^ test

**Fig 2:**
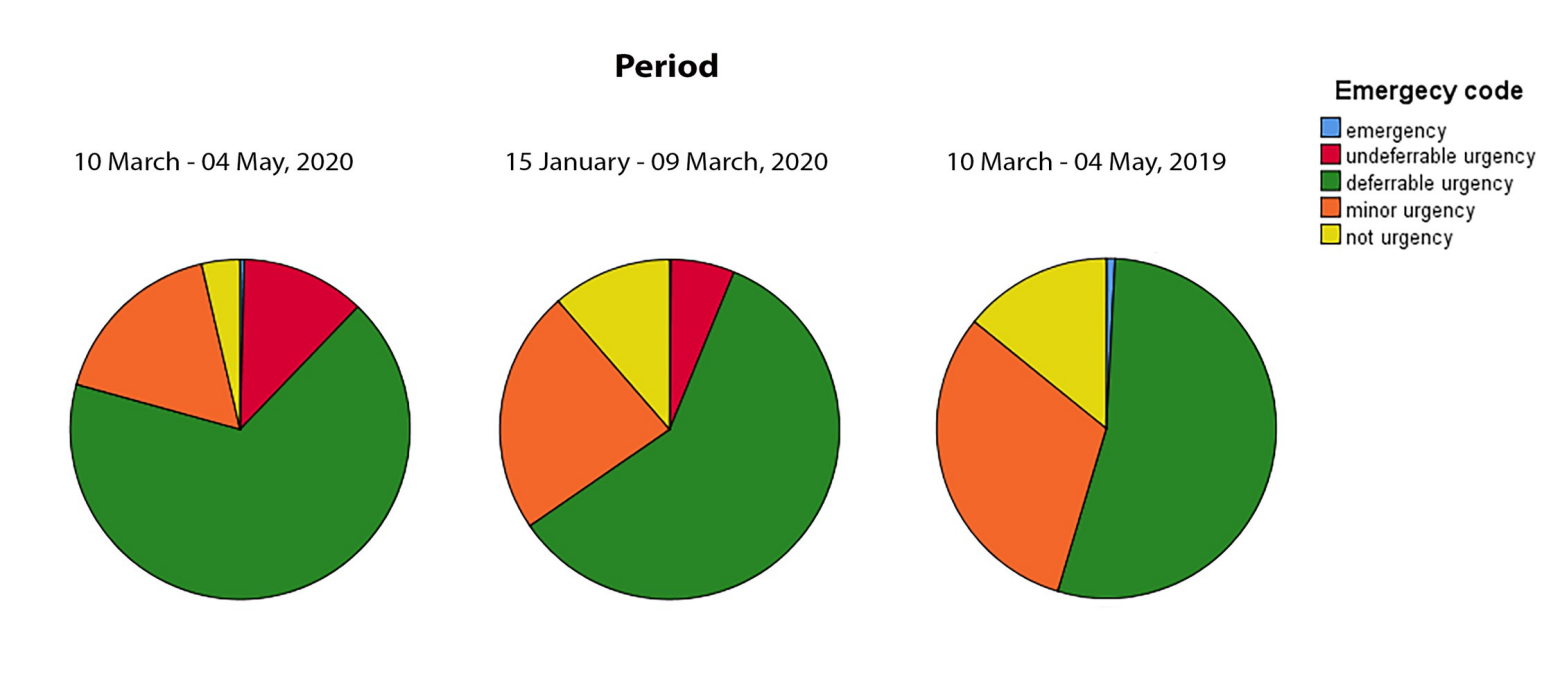
Number of visits categorized by emergency code. Pie charts display the number of visits performed at the Ophthalmological Emergency Department during the 2020 lockdown period (from March 10 to May 10), compared with the same period of 2019 and with the pre-lockdown of 2020 (from January 15 to March 09).

The percentage of minor and not urgency visits significantly decreased in lockdown period if compared with the same period (10 March– 04 May) of 2019. The percentage of not urgency visits significantly decreased when compared with the pre-lockdown period. In contrast, the percentage of non-deferrable urgency visits significantly increased in the lockdown period when compared with the other two periods (Table 2 and Fig 2). As undeferrable urgency visits have been considered all those conditions that could involve the anterior segment, the posterior segment or the eyeball, with a higher risk of permanent visual loss. Particularly, during the lockdown period, the main undeferrable conditions were: corneal abrasion with or without foreign body (27%), retinal detachment (13%), pseudomembranous conjunctivitis (13%), retinal tear (11%), acute angle-closure glaucoma (2%), endophthalmitis (2%), corneal ulcer (2%), branch retinal vein occlusion (2%).

**Table 2:** Number of visits categorized by emergency code.

Emergency code	10 MAR– 04 MAY 2020	15 JAN– 09 MAR 2020		10 MAR– 04 MAY 2019	
	Frequency	Frequency	*P value*^a^	Frequency	*P value*^b^
Emergency	1 (.4)	1 (.1)	.432	-	-
Undeferrable urgency	29 (11.8)	43 (6.0)	**.007**	8 (.8)	**< .001**
Deferrable urgency	165 (67.1)	421 (59.2)	.290	516 (53.8)	.053
Minor urgency	42 (17.1)	165 (23.2)	.101	299 (31.2)	**< .001**
Not urgency	9 (3.7)	81 (11.4)	**< .001**	136 (14.2)	**< .001**
Total	246	711		959	

Number of visits categorized by emergency code performed at the Ophthalmological Emergency Department during the 2020 lockdown period (from March 10 to May 10), compared with the same period of 2019 and with the pre-lockdown of 2020(from January 15 to March 09). The number of visits is presented in absolute and percentage values (between round brackets).

*P value*^a^ = lockdown period (10 Mar– 04 May 2020) compared with pre-lockdown period (15 Jan– 09 MAR 2020), χ^2^ test

*P value*^b^ = lockdown period (10 Mar– 04 May 2020) compared with the same period of 2019, χ^2^ test

**Fig 3:**
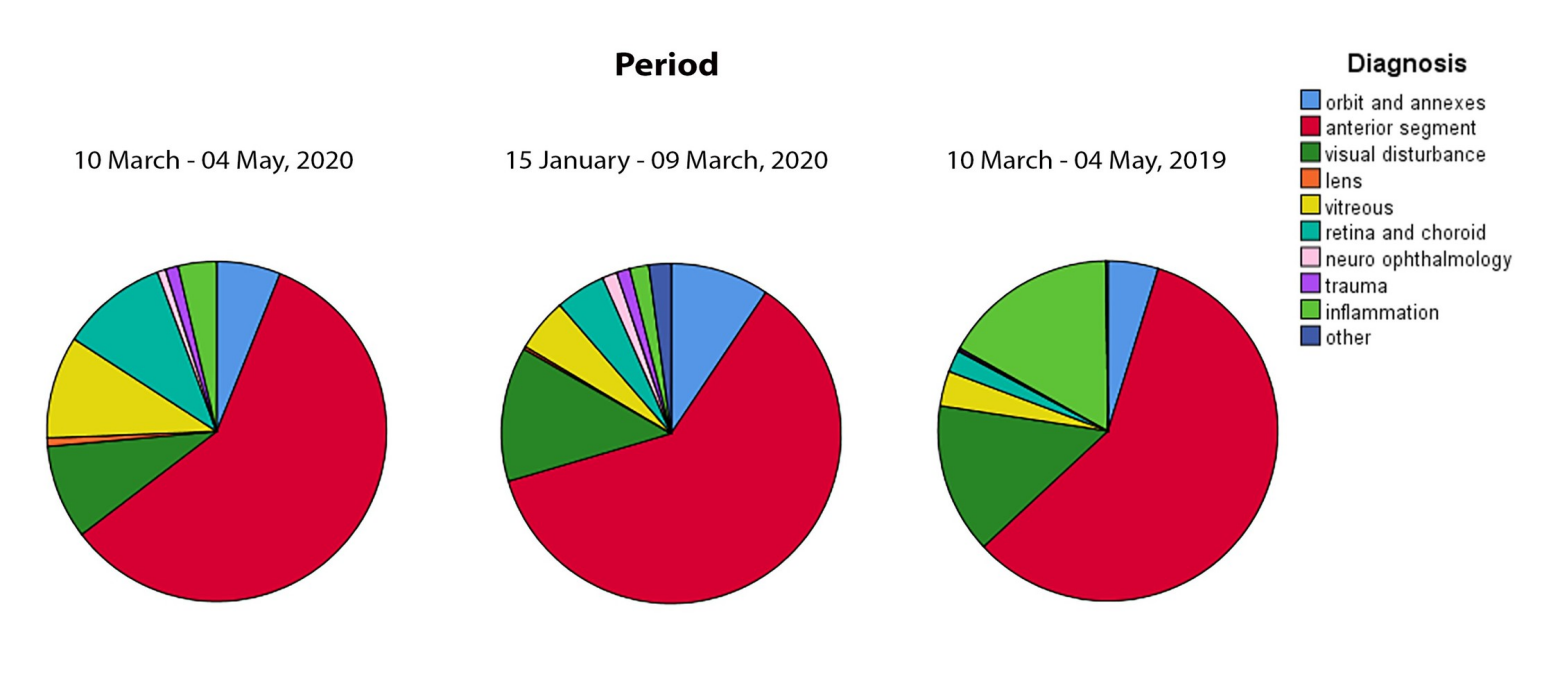
Number of visits categorized by diagnosis. Pie charts display the number of visits performed at the Ophthalmological Emergency Department during the 2020 lockdown period (from March 10 to May 10), compared with the same period of 2019 and with the pre-lockdown of 2020 (from January 15 to March 09).

Fig 3 shows the leading diagnoses categorized by period. The percentage of unspecified conjunctivitis significantly decreased in lockdown period if compared with the same period of 2019 (P <0.001, χ^2^ test) and with the pre-lockdown 2020 period (P = 0.002, χ^2^ test). We did not observe any change regarding the percentage of conjunctival hyperemia, foreign body, subjective visual disturbance, and corneal abrasion (all P > 0.05, χ^2^ test) between the considered periods (Table 3).

**Table 3:** Ophthalmological emergency department leading diagnoses categorized by period and ICD-9-CM.

10 MAR– 04 MAY 2020		15 JAN– 09 MAR 2020		10 MAR– 04 MAY 2019	
Diagnosis	Frequency	Diagnosis	Frequency	Diagnosis	Frequency
372.71 Hyperemia of the conjunctiva	29 (11.8)	372.30 Conjunctivitis, unspecified	117 (16.5)	372.30 Conjunctivitis, unspecified	242 (25.2)
360.50 Foreign body, magnetic, intraocular, unspecified	26 (10.6)	368.10 Subjective visual disturbance, unspecified	80 (11.3)	372.71 Hyperemia of the conjunctiva	160 (16.7)
368.10 Subjective visual disturbance, unspecified	22 (8.9)	918.1Corneal abrasion	75 (10.5)	368.10 Subjective visual disturbance, unspecified	132 (13.8)
918.1Corneal abrasion	19 (7.7)	372.71 Hyperemia of the conjunctiva	69 (9.7)	360.50 Foreign body, magnetic, intraocular, unspecified	88 (9.2)
372.30 Conjunctivitis, unspecified	19 (7.7)	360.50 Foreign body, magnetic, intraocular, unspecified	52 (7.3)	918.1 Corneal abrasion	71 (7.4)

Ophthalmological Emergency Department leading diagnoses categorized by period. Diagnosis were categorized using codes from the International Classification of Diseases, Ninth Revision, Clinical Modification (ICD-9- CM). Cases are reported as number and percentage values (between round brackets).

Diagnoses categorized by subclasses depending on the area of interest are displayed in Table 4. We observed that the percentage of vitreous diseases significantly increased in 2020 lockdown period if compare with the same period of 2019 (P < 0.001) and with the pre-lockdown period (P = 0.015). Similarly, the percentage of retinal and choroidal diseases significantly increased during the lockdown period if compared with 10 March- 20 May period of 2019 (P < 0.001) and with the pre-lockdown period (P = 0.004). The percentage of traumatic conditions had increased during the lockdown period when compared with 2019 (P = 0.028) while the percentage of diagnosis of ocular inflammation significantly decreased during the 2020 lockdown if compared with the same period of 2019 (P < 0.001)

**Table 4:** Number of visits categorized by diagnosis.

Diagnosis	10 MAR– 04 MAY 2020	15 JAN– 09 MAR 2020		10 MAR– 04 MAY 2019	
	Frequency	Frequency	*P value*^a^	Frequency	*P value*^b^
Anterior segment	144 (58.5)	434 (31)	0.729	559 (58.3)	0.971
Visual disturbance	22 (8.9)	91 (12.8)	0.147	137 (14.3)	0.049
Lens	2 (0.8)	2 (0.3)	0.267		-
Vitreous	24 (9.8)	36 (5.1)	**0.015**	32 (3.3)	**<0.001**
Orbit and annexes	15 (6.1)	67 (9.4)	0.137	46 (4.8)	0.431
Retina and choroid	25 (10.2)	34 (4.8)	**0.004**	20 (2.1)	**<0.001**
Neuro ophthalmology	2 (0.8)	10 (1.4)	0.475	1 (0.1)	**0.047**
Trauma	3 (1.2)	9 (1.3)	0.955	2 (0.2)	**0.028**
Inflammation	9 (3.7)	13 (1.8)	0.108	160 (16.7)	**<0.001**
Other	-	15 (2.1)	-	2 (0.2)	**-**
Total	246	711		959	

Number of visits categorized by diagnosis performed at the Ophthalmological Emergency Department during the 2020 lockdown period (from March 10 to MaY 10), compared with the same period of 2019 and with the pre-lockdown of 2020(from January 15 to March 09). The number of visits is presented in absolute and percentage values (between round brackets).

*P value*^a^ = lockdown period (10 MAR– 04 MAY 2020) compared with pre-lockdown period (15 Jan– 09 MAR 2020), χ^2^ test0.971

*P value*^b^ = lockdown period (10 MAR– 04 MAY 2020) compared with the same period of 2019, χ^2^ test

We also retrieved the number of ophthalmological consultations in the periods considered in this report. Our emergency department demanded ophthalmological consultation in 25 cases during the 2020 lockdown period in contrast with the 149 cases (94 males, 63.1%) of the same period of the 2019 and with the 129 cases of the pre-lockdown period of 2020. Mean age of patients requiring ophthalmological advice was 33.28 ± 27.96 during March 10—May 04, 2019.

Leading conditions for which ophthalmological consultation was sought were cranial trauma (3 cases, 12%), headache (3 cases, 8%), diplopia (2 cases, 8%), subjective visual disturbance, unspecified (2 cases, 8%); fracture of the orbital floor (2 cases, 8%) during the 2020 lockdown period; unspecified conjunctivitis (21 cases, 14.1%), headache (18 cases, 12.1%), cranial trauma (17 cases, 11.4%), diplopia (11 cases, 7.4%), unspecified contusion of the eye (8 cases, 5.4%) during the March 10 –May 04, 2019, period; headache (24 cases, 18.6%), cranial trauma (20 cases, 15.5%), unspecified conjunctivitis (15 cases, 11.6%), subjective visual disturbance, unspecified (8 cases, 6.2%), contusion of the eye (6 cases, 4.7%) during January 15- March 09, 2020, period. We did not observe any change regarding the percentage of cranial trauma, headache and diplopia (all P > 0.05, χ^2^ test) between the considered periods.

## Discussion

This study aimed to compare patient’s accesses to an emergency department of a tertiary referral center before and during the lockdown for pandemic COVID-19.

As observed for previous epidemics [9], we stated a dramatic reduction of visits. We observed a reduction of 65.4% if compared with the pre-lockdown period and a reduction of 74.3% if compared with the same period of 2019. In the early 2000s during the SARS epidemic in Taiwan, Chang and coworkers [9] reported a reduction of health services expenditures, particularly for ambulatory and inpatient services. This reduction started with the epidemic outbreak and continued even after the World Health Organization took Taiwan off the list of SARS-affected countries. The authors suggested that the fear of SARS significantly affected people’s healthcare-seeking behavior and that this fear seriously influenced their access to medical care [9].

In the first month of lockdown, a reduction of patients’ access to hospitals was highlighted by some papers [7, 8], even for life-threatening conditions, such as myocardial infarction. Lazzerini and coworkers [8] published data about some pediatric patients during the first month of the pandemic. Because of the risk of being exposed, some children have experienced serious complications or even death. Their paper emphasized that delay in access to care could be more dangerous than the risk of being exposed.

In the same period, even for ophthalmological conditions, we observed a decrease in ocular traumas. Pellegrini and coworkers [11] reported a significant reduction of ophthalmological emergency department visits during the first 6 weeks of lockdown compared with those of the same period of 2019. Particularly, they pointed out a reduction of infectious conjunctivitis among children and adolescents, maybe due to the social distancing and the school’s closure and a reduction of non-urgent conditions. The same authors described also a reduction of serious emergencies such as retinal detachments with a higher risk of visual impairment. In this case, the authors hypothesized patients’ reluctance to be exposed to SARS-Cov-2 in hospitals [11].

In our hospital, we detected a significant decline of “not urgency visits” compared with those of pre-lockdown and those of 2019 and a significant reduction of “minor urgency visits” compared with those of 2019. Usually, are defined as “minor urgent” conditions such as conjunctivitis, conjunctival hyperemia, unspecified subjective visual disturbance and unspecified eye inflammations.

On the other hand, “Undeferrable urgency visits” significantly increased. We classify as undeferrable urgencies all those conditions that can cause a sudden or progressive visual loss and, therefore, force the patient to access to an ophthalmological emergency department. These conditions could involve the anterior segment (e.g. herpetic corneal infections, anterior uveitis, acute angle-closure glaucoma, trauma), the posterior segment (e.g. posterior uveitis, branch and central vein occlusion, branch and central artery occlusion, retinal tear with or without retinal detachment, trauma) or the eyeball (endophthalmitis).

As for traumas, analyzing patients’ medical charts before and during the lock-down, an increased number of domestic accidents emerged. This raise may be related to the availability of time to do chores around the house and the yard. Even for the ophthalmological consultations, we have noted a significant reduction. Our ophthalmological emergency department is responsible for consultancy activity. We observed a drastic fall in our services with a total of 25 consultancies during the lockdown. Compared to previous periods, the leading causes of consultation were undeferrable conditions: trauma. This lower consultancy activity may be related to the suspension of elective hospitalizations and surgeries. Moreover, most wards were converted into ‘COVID Units’ to face the emergency.

Considering our results, no difference in gender and age was remarked before and during the lock-down period. These findings were consistent with the consideration that there is no age- or gender- related distinction regarding patients seeking for ophthalmological emergency department despite the pandemic. Patients of all ages appear to be susceptible to eye emergencies.

Our data overall showed a dramatic fall of emergency patient’s accesses during the lockdown, although ophthalmological emergency department was open for acutely ill patients following all the anti-SARS-Cov-2 security measures. In accordance with previous studies [7, 8, 11], a possible explanation of these findings could be the patient’s fear of being infected. During the pandemic, people’s priority was preserving life [5]. Sight-threatening conditions together with life-threatening ones were considered less important. The same fear already described in the past for the SARS epidemic [9].

We supposed that patients with “minor and not urgency conditions” preferred being visited in private ophthalmology centers regarding this structure as safer because there were no hospitalized COVID-19 patients. However, this hypothesis raises other questions: which are the real ophthalmological emergencies? Is it right to use first aid for “minor and not urgency visits”? These considerations highlighted one of the main problems of Italian health service. Our healthcare system [13] makes the right to health accessible to all citizens, without discrimination based on income, gender or age. Nevertheless, the number of hospitals and healthcare personnel are often inadequate to the burden of patients. Thus, to avoid long waiting lists patients misuse emergency services seeking for first or chronic follow-up visits. Maybe, the spread of telemedicine together with an increased number of physicians both in the hospital and community settings, may allow reducing the overcrowding in our emergency departments [14–17], hence warranting safety for low-acuity patients, their family members and healthcare professionals [18]. Finally, the Italian National Health Care System should strengthen the role of public health who display a central role for research and patient care. One of the main limitations of the present study is its retrospective nature, moreover, we performed only quantitative analysis of data and diagnosis were categorized using codes from the International Classification of Diseases, Ninth Revision, Clinical Modification (ICD-9-CM). A dedicated classification system drafted by the main International Ophthalmology Societies would be desirable especially for ultra-specialized disciplines such as ophthalmology. Another relevant consideration is that the emergency code is established by each ophthalmologist based on its clinical judgement. Moreover, the codification system in emergency, undeferrable urgency, deferrable urgency, minor urgency and not urgency could be improved because the code is related with the waiting time. However, in ophthalmology there aren’t life threatening condition for which immediate intervention is required.

## Conclusions

In conclusion, our data depict an insight into the real clinical practice to derive information for improving patient’ management both in an ordinary and extraordinary situation such as the recent SARS-Cov-2 pandemic.
